# ADDRESSING SYSTEMIC RACISM ACROSS LONG-TERM SERVICES AND SUPPORTS

**DOI:** 10.1093/geroni/igac059.595

**Published:** 2022-12-20

**Authors:** Shekinah Fashaw-Walters, Tetyana Shippee, Jasmine Travers

**Affiliations:** University of Minnesota School of Public Health, Minneapolis, Minnesota, United States; University of Minnesota, Minneapolis, Minnesota, United States; NYU, New York City, New York, United States

## Abstract

Long-term services and supports (LTSS) are some of the most racially segregated healthcare services in the U.S. today. Marginalized users (e.g., Black and Latino older adults) have disproportionate access to high-quality care and subsequently report poorer health outcomes when compared to White users. It is important to acknowledge racism as a fundamental cause of these inequities to LTSS access. As the U.S. works to expand LTSS, it is critical to diversify and strengthen the LTSS workforce, increase Medicaid reimbursements along with efforts to improve accountability and transparency, reconsider payment models and the use of public reporting, improve quality metrics, implement effective support systems for patients of color, expand access to care, and increase promotion of integrated care. Health equity researchers, Drs. Tetyana Shippee, Shekina Fashaw-Walters, and Jasmine Travers will share 7 actionable evidence-based recommendations for LTSS policy change aimed to dismantle racism and advance health equity.

